# Signal Transduction Pathways Activated by Innate Immunity in Mast Cells: Translating Sensing of Changes into Specific Responses

**DOI:** 10.3390/cells9112411

**Published:** 2020-11-04

**Authors:** Zyanya P. Espinosa-Riquer, Deisy Segura-Villalobos, Itzel G. Ramírez-Moreno, Marian Jesabel Pérez Rodríguez, Mónica Lamas, Claudia Gonzalez-Espinosa

**Affiliations:** Departamento de Farmacobiología, Centro de Investigación y de Estudios Avanzados (Cinvestav), Unidad Sede Sur. Calzada de los Tenorios No. 235, Col. Granjas Coapa, Mexico City 14330, Mexico; espinosariquer@gmail.com (Z.P.E.-R.); deisy.segura@cinvestav.mx (D.S.-V.); itzel.irm@gmail.com (I.G.R.-M.); mjperezr@cinvestav.mx (M.J.P.R.); mlamas@cinvestav.mx (M.L.)

**Keywords:** mast cells, signal transduction, innate immunity

## Abstract

Mast cells (MCs) constitute an essential cell lineage that participates in innate and adaptive immune responses and whose phenotype and function are influenced by tissue-specific conditions. Their mechanisms of activation in type I hypersensitivity reactions have been the subject of multiple studies, but the signaling pathways behind their activation by innate immunity stimuli are not so well described. Here, we review the recent evidence regarding the main molecular elements and signaling pathways connecting the innate immune receptors and hypoxic microenvironment to cytokine synthesis and the secretion of soluble or exosome-contained mediators in this cell type. When known, the positive and negative control mechanisms of those pathways are presented, together with their possible implications for the understanding of mast cell-driven chronic inflammation. Finally, we discuss the relevance of the knowledge about signaling in this cell type in the recognition of MCs as central elements on innate immunity, whose remarkable plasticity converts them in sensors of micro-environmental discontinuities and controllers of tissue homeostasis.

## 1. Introduction

The immune system (IS) has been defined as a distributed and autonomous entity, since it is composed of many distinct and numerous cells and seems to react without an apparent director [1]. Accordingly, one of the main design principles of the IS refers to the dynamic engagement of different cells, which act only briefly and then are replaced by others that respond to the new microenvironment [2]. Thus, tissue resident immune cells, which sense danger signals and translate those stimuli into the production of a specific cocktail of pro-inflammatory mediators, determine the timing and shaping of a given specific innate immune response. The knowledge of specific signal transduction pathways operating in each one of those cell lineages is important to understanding the mechanism by which the IS awakens and is ultimately organized to deal with infections, tumors or tissue damage. 

The mast cells (MCs) are the main players of inflammatory reactions and, in concert with other cells, shape specific tissue responses to infection and damage. In the embryo, they are generated at the yolk sac, from where they colonize distinct tissues. In late phases of development, those populations are mostly replaced by definitive, hematopoietic, stem cell-derived progenitors that, at least in the skin, form clonal colonies [3,4,5]. Located mainly in interface tissues that are in contact with the environment, MCs have been called the sentinels of innate immunity, executing rheostatic functions [6,7,8]. Although they are better known by their role on allergic responses, research performed during the last twenty-five years has clarified its involvement in innate immunity. Indeed, they can be activated by a number of pathogens and damage- associated molecular patterns (PAMPs and DAMPs) that are recognized by pattern recognition receptors (PRRs) [9,10]. Also, MCs are activated by irritant chemicals, bacterial products and peptides because they express several members of the Mas-related G protein-coupled receptors (MRGPRs) family. In the course of an inflammatory reaction, MCs sometimes act in altered environments (such as highly oxidant and hypoxic conditions). The activation of MCs receptors engages specific signaling networks and leads to the selective release of soluble or exosome-contained inflammatory mediators that not only initiate local and rapid reactions, but also sustain and shape long-term responses that contribute both to tissue repair and chronic damage. 

Here, we present the recent evidence regarding to the description of the main signal transduction pathways that connect innate immunity receptors to the secretion of soluble or exosome-contained pro-inflammatory mediators by MCs, making emphasis in the participation of canonical molecules with distinctive functions on this cell type, the mechanisms of negative control of PRR activation and the effect of environmental conditions, such as hypoxia, on MC signaling. 

## 2. Dynamic Activation of MCs in Innate Immunity Reactions

The participation of MCs on type I anaphylactic, allergic reactions after the crosslinking of the high affinity IgE (FcεRI) receptor has been largely demonstrated [11] whereas their role on innate immunity protective reactions was formally recognized around twenty-five years ago [12,13]. Research on the signaling pathways activated by the FcεRI receptor by monomeric IgE and by IgE/antigen (Ag) complexes has identified the main molecular mechanisms leading to the rapid secretion of pre-formed mediators (anaphylactic degranulation) and de-novo synthesized lipids and cytokines in this cell type [14,15]. In contrast, the signaling pathways activated by PRRs in this cell type have remained elusive.

**Figure 1:**
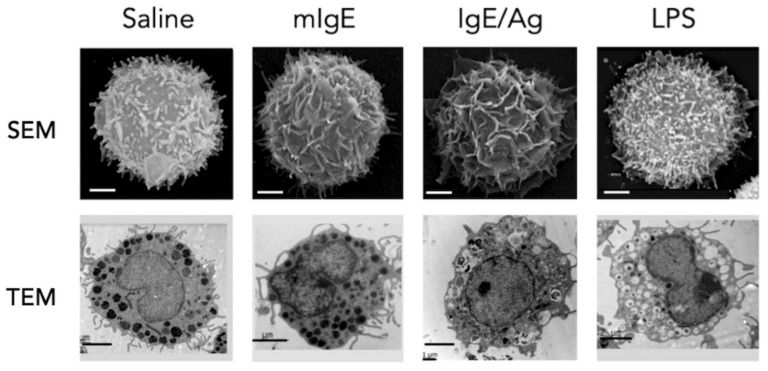
Plasticity on MCs responses elicited by distinct stimuli. Mature bone marrow derived mast cells (BMMCs) were treated with saline solution (Saline), monomeric IgE (mIgE, 100 ng/mL), IgE/Ag complexes (3 ng/mL) and *Escherichia coli* LPS (B06 serotype, 500 ng/mL) for optimal times to observe main ultrastructural changes (saline and mIgE 24 h; IgE/Ag 2 min and LPS 30 min). Samples were next processed for scan electron microscopy (SEM) and transmission electron microscopy (TEM). Presence of mIgE causes an increase on BMMCs mature granules without detectable degranulation; IgE/Ag complexes trigger anaphylactic degranulation and LPS addition induce piecemeal degranulation in IgE-sensitized BMMCs. Pictures were obtained by Marian J. Pérez-Rodríguez and Alfredo Ibarra-Sánchez (Cinvestav).

Biochemical and cell biology approaches have demonstrated that, depending on the stimulus they receive, MCs suffer important morphological changes leading to specific processes of secretion, mainly classified in three major types: anaphylactic degranulation, piecemeal degranulation and constitutive compound secretion [16]. Anaphylactic degranulation is characterized by the rapid release of pre-formed compounds, such as histamine, serotonin, and Tumor Necrosis Factor alpha (TNF-α) and occurs by the fusion of pre-formed granules to the plasma membrane in a process highly dependent on significant calcium mobilization and cytoskeletal re-arrangements. On the other hand, piecemeal degranulation is associated to the gradual emptying of the secretory granules, without an evident granule-to-plasma membrane fusion. Finally, secretion by the constitutive pathway is characterized by the production of mediators that are newly synthesized and released directly from vesicles that are generated in the trans-Golgi network. To date, evidence indicates that PRR stimulation of MCs does not lead to anaphylactic degranulation, but activates signaling networks leading to piecemeal degranulation and cytokine secretion by the constitutive pathway (Figure 1). 

Information from different cellular models of MCs indicates that main PRRs expressed on this cell lineage are members of the Toll-like (TLR), the NOD-like and the RIG-I-like families of receptors. Also, MCs express members of the C-type Lectin family of receptors and Mas-related G protein-coupled receptors [17]. Activation of those molecules activate particular canonical and non-canonical signaling cascades coupled to the production of mediators of inflammation that can be secreted in a soluble form or contained in exosomes to finally generate a specific response in a given tissue. In the following sections, we present the main signal transduction cascades activated by PRRs in the MCs and discuss the effects of environmental conditions (such as hypoxia) on MCs secretory responses.

## 3. Toll-Like Receptors (TLRs)-Dependent Activation of MCs

TLRs are the most studied innate immune receptors. They are type 1 transmembrane glycoprotein receptors located in the plasma membrane or endosomes of a number of immune cells and belong to the interleukin (IL)-1 receptor/TLR superfamily of proteins. They have an extracellular leucine-rich repeat (LRRs) domain, which mediates recognition of PAMPs and DAMPs, a transmembrane domain and, finally, a cytosolic or intracellular Toll/IL-1R-like (TIR) domain required for the activation of early phases of downstream signaling pathways [17,18]. TLRs recognize a wide range of molecular patterns associated with bacteria, viruses, and other microorganisms, as well as different factors that result from tissue damage [17,18,19]. 

Some published reviews have focused their attention on the presence of TLR receptors in distinct MCs populations [20,21], and recent evidence indicates that TLR-dependent activation of this cell type presents distinctive and particular features. 

The pattern of expression of TLRs on the MCs depends on the experimental preparation under study. Several groups have analyzed murine, human, and mast cell lines to establish which TLRs are present on these different cellular models. Murine MCs express TLRs1–4 and 6–9 mRNAs [21,22,23,24,25,26,27,28]. In contrast, in purified MCs from the peritoneal cavity, no TLR was detected in a proteome analysis [29]. Expression of TLRs in human MCs has been controversial, since some studies have demonstrated the presence of TLRs1-10 with the exception of TLR8, although other groups were not able to detect TLR1, 4, 6, or 9 [30,31,32,33,34,35]. In line with this, analysis of mast cell lines such as LAD2 (Laboratory of Allergic Diseases 2), HMC-1 (Human Mast Cell line 1) and MC/9 have also produced contrasting results [22,24,33,34,35,36]. In a more recent work, the analysis of TLRs in MCs purified from the peritoneal cavity of rats (connective tissue MCs) showed not only the expression, but also the location of mRNAs and proteins of TLR2, TLR3, TLR4, TLR5, TLR7, and TLR9 in the cell. TLR4 was identified in the cell surface while TLR5 and TLR7 were only observed in the interior of the cells. In contrast, TLR2, TLR3, and TLR9 were identified in both, the plasma membrane and intracellular compartments, being the nuclear envelope and/or perinuclear area the preferential location for TLR3 and TLR9 [37]. Moreover, the authors described that the antimicrobial peptide cathelicidin (LL-37) can modify TLRs expression and distribution [37], suggesting that other external stimulus or environmental factors could modify the expression and location of these receptors, thereby altering MCs’ innate response. 

Another receptor of the IL-1 superfamily has been recently described in MCs. This suppressor of tumorigenicity 2 (ST2) receptor shares most of the signaling system of the TLRs, however it is activated by IL-33, an alarmin that is produced in response to infection or injury [38]. For this reason, it will be discussed in the section pertaining to the stress-induced activation of MCs.

### 3.1. TLR4-Dependent Activation of MCs

The TLR4 receptor has been extensively studied due to its relevance on the innate immune response against different insults and its broad pattern of expression that include microglia, macrophages, dendritic cells (DCs) and MCs. It is classically activated by bacterial lipopolysaccharide (LPS, endotoxin), the main component of Gram-negative bacteria membrane, and its activation has been related to protective reactions and chronic and acute inflammatory disorders. TLR4 can be also activated by DAMPs such as the high mobility group box 1 (HMGB1) protein, an alarmin secreted by damaged cells [39]—this will be discussed in a later section. The canonical TLR4 ligand recognition process, the signaling system and the triggered responses, have been described mainly in macrophages, macrophage-like cell lines, freshly isolated monocytes and DCs. Ligand recognition seems to initiate when the LPS binding protein (LBP) presents LPS to the co-receptor CD14, which helps to engage this molecule to a complex formed by TLR4 and the myeloid differentiation protein 2 (MD-2). Then, after a conformational change that occurs on the TIR domain, a receptor multimer composed of two TLR4-MD2-LPS complexes, starts the downstream signaling pathway. Triggering of TLR4 involves the MyD88- and the TIR-domain-containing adapter-inducing interferon-β (TRIF)-dependent pathways, with the subsequent activation of AP-1, interferon-regulatory factor (IRF)5, NFĸB and IRF3 transcription factors, and, finally the de novo synthesis of cytokines (including TNF-α and IL-6) and other inflammatory mediators [20]. The mechanisms of activation and the study of the signaling pathways activated by TLR4 receptor in MCs have been of special interest in recent years. This is due to the fact that they are one of the first cell populations to respond to infections or tissue damage given their strategic location at the interface between host and environment, and they also present specific features of inflammatory mediators’ production, storage and release. Moreover, MCs have been recently associated with protective immune reactions against Gram-negative bacteria [12,13,20,23,40,41], which demonstrates that these cells are required for host defense in animals and humans [31,42].

Several MCs preparations have been studied to determine the features of the TLR4 receptor and its signaling cascades leading to the production of inflammatory mediators. Regarding LPS recognition processes, the expression of CD14 co-receptor and of the MD2 protein is controversial. It has been shown that, although most murine bone marrow-derived mast cells (BMMCs) from different mice strains, and MC/9 cells express MD2, the presence of CD14 on the plasma membrane is not detectable [22,43,44,45]. However, mRNA of the latter has been identified in BMMCs from BALB/c [25], C57BL6/J, and R6/1 mice [46,47]. Using human intestinal mast cells (hiMCs), Brenner and cols. [48] found that lack of CD14 protein expression on these cells make them tolerant to Gram negative microbiota, but they can still recognize and eliminate potential pathogens due to the participation of soluble CD14 (sCD14) distributed by other cells [48].

The MyD88-dependent signaling triggered with LPS initiates in the cell membrane in macrophages and DCs [21,49]. MyD88 is recruited via the adaptor TIRAP and it interacts with the family of interleukin-1 receptor associated kinases 1 and 4 (IRAK1 and IRAK4) [21]. Very little is known about IRAK roles on TLRs signaling in MCs. In MC/9 cell line, LPS was shown to activate IRAK1, suggesting that this kinase is important in signaling from TLR4 [50]. Moreover, by using IRAK1 deficient (*Irak^−/−^)* BMMCs, it has been shown that absence of this protein prevents the response of MCs to LPS [21,51]. Information about the expression of IRAK4 in MCs is scarce, however, it seems to participate just as in other immune cells after TLR4 activation. In BMMCs, activation of MyD88 also leads to the function of Src kinases Lyn and Fyn which regulate downstream effectors [46,52]. Lyn promotes TNF receptor associated factor 6 (TRAF6) ubiquitination and, in consequence, activation of the transforming growth factor-β activated kinase 1 (TAK1) [52], while Fyn kinase activation acts as a negative regulator of TLR4 signaling pathway by increasing phosphorylation of the protein phosphatase 2A (PP2A). In this form, Fyn-dependent activation of PP2A leads to the inhibition of TLR4-dependent activation of protein kinase Cα/βII (PKCα/βII), CamKII and p70S6K, limiting excessive cytokine production after LPS stimulation [46]. 

In distinct immune cells TAK1 activation results in TLR4 receptor signaling cascade bifurcating into the IĸB kinase (IKK)-dependent and the MAPKs-dependent pathways. In different studies on MCs, IKK has been found to be active as early as 15 min after LPS addition [47,53,54,55], and its substrate, IĸB-α/β, is also phosphorylated [40] in a TLR4-dependent manner [23,40]. The phosphorylation of IĸB, leading to its posterior degradation, induces NFκB nuclear translocation for the initiation of inflammatory-related gene transcription. Activation of NFκB in MCs after TLR4 triggering has been described in diverse studies. Using C57BL6/J derived BMMCs or HMC-1 cells, TLR4 stimulation has shown to induce p65 phosphorylation [47,54,55] and NFκB nuclear translocation [56,57]. Interestingly, endotoxin-induced activation of IKK seems to be involved in exocytosis of TNF-α but not in the transcription *tnfα* gene, since the IKK inhibitor BAY117085 prevented TNF-α secretion but not mRNA expression in LPS-stimulated BMMCs [47,53].

Besides the IKK-dependent signaling pathway, different studies have demonstrated that the MAPKs-dependent branch is also important in TLR4 receptor signaling. Originally, p38 and JNK were thought to play a more important role than ERK1/2 in TLR4 receptor signal transduction [24,50,58], however, recent studies have demonstrated that LPS-induced ERK1/2, p38 and JNK phosphorylation [47,54,55,56] is essential for MCs response to LPS, as specific inhibitors of these proteins prevented endotoxin effects on those cells [53,54]. AP-1 and ATF2 transcription factors are activated as a result of MAPKs pathway stimulation, as demonstrated in MC/9 MCs by the increase of ATF-2 and c-Jun activation in response to LPS [50]. Additionally, BMMCs have shown to increase *c-fos* mRNA accumulation after TLR4 triggering, which depends on LPS-induced ERK1/2 (and its associated transcription factor ELK-1) phosphorylation [47].

Aside of MyD88 pathway activation, phosphatidylinositol 3-kinase (PI3K) seems to be important on TLR4 signaling in MCs. Signal from PI3K activation is quite important for MCs development, homeostasis and secretion of inflammatory mediators. It is activated downstream of many membrane receptors such as FcεRI, the receptor for Stem Cell Factor (c-KIT) and TLRs. By using the PI3K inhibitors wortmannin [53] and LY294002 [59], BMMCs were shown to respond less to LPS as demonstrated by the prevention of TNF-α [53,59], IL-6 and IL-1β [59] secretion. Some other studies have shown contrasting results regarding the participation of PI3K on IL1-β secretion after TLR4 triggering [60], although this difference has not been further studied. The participation of PI3K in the endotoxin-induced response seems to depend on the cells studied as corroborated with MC/9 cells, where AKT, a downstream effector of PI3K, was not phosphorylated in response to LPS [50]. 

As mentioned before, TLR4 signaling proceeds from the MyD88-dependent signaling as well as from the TRIF-dependent pathway in macrophages, monocytes and DCs. The latter signal occurs from early endosomes after TLR4 receptor endocytosis [44], a process that has been demonstrated to be dependent on the expression of CD14 [61]. TRIF activation couples to the adaptor molecule TRAM and culminates with the activation of the transcription factor IRF3 and the production of IFN-β. It also leads to a delayed NFκB and AP-1 activation [45,62]. In addition to CD14 expression, the existence of a TRIF-dependent pathway has been controversial in MCs. Originally, it was demonstrated that TLR4 is not internalized after stimulation of this cell type [63] and that TRIF signaling, IRF3 activation and the subsequent production of IFN-β is absent in BMMCs and peritoneal MCs from C57BL6/J mice in response to endotoxin stimulation [45]. This supports the idea that the lack of this pathway in MCs prevents excessive cytokine production to avoid continue inflammatory responses in sites where these cells are in constant contact with Gram-negative bacteria [45,48]. 

Endocytosis of TLR4 in MCs, however, does seem to be necessary for the activation of the MyD88-dependent signaling branch of the cascade, since it was required for ERK1/2 phosphorylation, *c-fos* accumulation and expression of TNF-α mRNA [47]. Utilizing the dynamin inhibitor dynasore and confocal microscopy, it has been recently shown that TLR4 receptor is primarily located in the cytosol of BMMCs and that, after LPS addition, it is translocated to perinuclear locations in a process that requires the multifunctional transport protein Huntingtin [47]. Data related to intracellular location of TLR4 receptor in BMMCs agree in studies using different MCs models [31,45,56]. The differences in LPS-induced TLR4 receptor internalization and the absence of TRIF-dependent pathway in MCs could be related to cell culture characteristics (IL-3 source, presence of stem cell factor (SCF) and other cytokines) or the nature of the IgE used for MCs sensitization. Indeed, these may change the phenotype of MCs and, therefore, alter the expression, location and activity of some receptors and signaling cascades [64].

It has been shown that the TLR4-TRIF-dependent pathway, in macrophages, depends on the PLC-γ 2-IP_3_-Ca^2+^ cascade where the release of intracellular Ca^2+^ mediates TLR4 trafficking and activation of IRF3 [65]. Because LPS-induced TNF-α secretion in BMMCs depends on intracellular Ca^2+^ release (as BAPTA inhibits this response) [53], it is possible to hypothesize that LPS-induced increase of calcium in BMMCs could contribute to endocytosis of TLR4 receptor and to a possible low-level activation of TRIF-dependent pathway.

Compared with the FcεRI receptor, activation of TLR4 signaling in MCs is considered a low intensity stimulus [66] that causes cytokine release but not promotes the fusion of cytoplasmic pre-formed granules with plasma membrane. However, some in vivo studies have shown that LPS can induce robust MCs degranulation [67,68] in a TLR4- and MyD88-dependent manner [67]; these discrepancies suggest that microenvironment, culture conditions or the origin of the MCs preparation could change MCs responses to TLR4-triggering. It has been consistently reported that TLR4 signaling causes a release of a wide range of inflammatory mediators, that include TNF-α, IL-5, IL-6, IL-10, IL-13, IL-1β, GM-CSF, CCL1, CCL2, CCL3, and CXCL2, as well as leukotrienes and other eicosanoids [66]. In particular, it has been described that endotoxin stimulation induces cytokine secretion through the constitutive and the piecemeal secretion pathways. Constitutive secretion is used by newly synthesized mediators, while preformed cytokines will preferentially be released by piecemeal degranulation [14,20,69]. Some cytokines, such as TNF-α, use both types of exocytosis since they can be synthesized de novo or preformed and stored in secretory granules of MCs [53,54,69]. In macrophages and other immune cells, the study of molecules of the signaling system that induce the activation of transcription factors, expression of cytokine genes and constitutive secretion of cytokines, encompasses the understanding of the canonical inflammatory response as a consequence of TLR4 triggering. However, in MCs, the analysis of the mechanisms that regulate secretion, not necessarily the de novo synthesis of inflammatory mediators, is also relevant.

**Figure 2:**
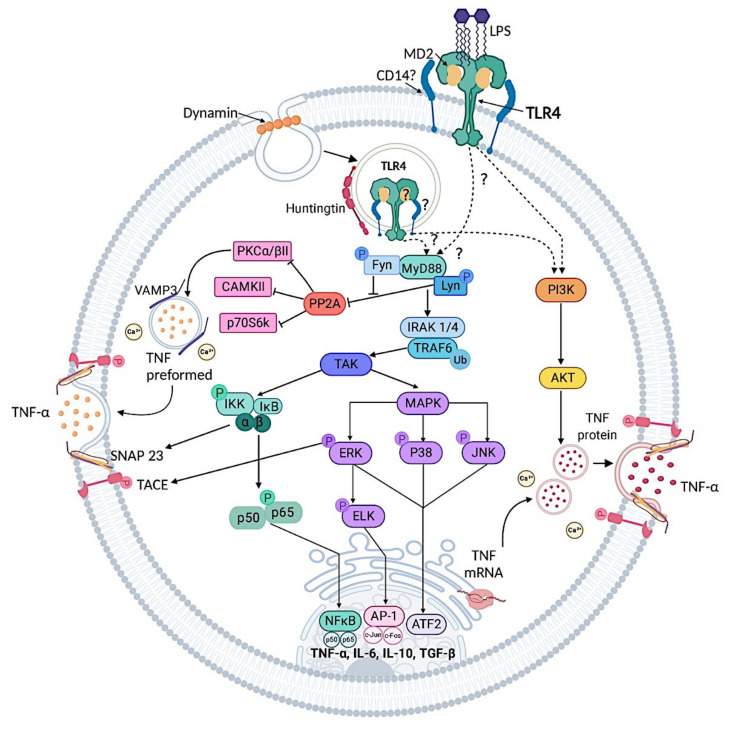
Toll-like 4 receptor signaling in MCs. TLR4 triggering occurs after TLR4/MD2-complex dimerization. Although CD14 has been detected in MCs at mRNA level, its function in LPS recognition has not been completely elucidated. Signaling from TLR4 receptor on MCs could occur from PM or endosomes, however, internalization-dependent and -independent activation of some elements of the cascade remains to be proven (see text for more references). MyD88-dependent pathway requires the participation of the Src kinases Fyn and Lyn. Lyn induces the activation of IRAK1/4, which in consequence activates TRAF6 and TAK1 proteins; on the other hand, Fyn controls PP2A/PKCα/βII axis that negatively regulates signaling. At TAK1, signal bifurcates into the IKK- and the MAPKs- dependent pathways. IKK activates NFĸB transcription factor, which translocates to the nucleus to start production of inflammatory cytokines. On the other hand, phosphorylation of MAPKs activates AP-1 and ATF2, which contribute to inflammatory reactions. Signaling cascades are also linked to secretory control as PKCα/βII regulates VAMP3+ vesicles, IKK phosphorylates SNAP-23, and ERK1/2 activates TACE, all of which control TNF-α release. Other molecules have been described to participate in TLR4 signaling such as Ca^2+^, and PI3K/AKT cascade, which also contribute to TNF-α secretion. Figure created with Biorender.com.

Studies on the TLR4 receptor signal transduction system on MCs and endotoxin-dependent TNF-α release have led to the description of new molecular players that connect that receptor signaling to the secretion of cytokines, and not only to cytokine gene transcription [46,53,54]. In line with this, it has been demonstrated that, in BMMCs, TNF-α is contained in vesicle-associated membrane protein 3 (VAMP3) positive vesicles that accumulate in the plasma membrane in response to LPS. Further, Fyn kinase regulates fusion of those vesicles through the modulation of PP2A/PKC axis to limit TNF transcription and also its release [46]. Moreover, different studies in MCs have demonstrated that IKK and ERK1/2 branches not only contribute to the activation of transcription factors, but also regulate secretion per se [53,54]. By using BMMCs from C57BL6/J mice, Madera-Salcedo and cols. demonstrated that after LPS stimulation, activation of IKK, in addition of inducing IκB activation, leads to the phosphorylation of the synaptosomal-associated protein 23 (SNAP23), which is important for vesicle fusion with the plasma membrane and TNF-α release [53]. On the other hand, ERK1/2 activation was also shown to be associated with the phosphorylation of the protein TACE (tumor necrosis factor alpha-converting enzyme), which is essential for TNF-α maturation and secretion [54] (Figure 2). 

Interestingly, it has been documented that LPS (via TLR4 receptor) can amplify MCs responsiveness to other stimuli. An example of the latter is the study of the effect TLR4 triggering on FcεRI-induced responses, in which LPS has been proved to enhance degranulation of RBL-2H3 and mouse peritoneal MCs through the increase of Ca^2+^ entry [70]. Accordingly, TLR4 triggering enhances antigen-dependent degranulation, leukotriene production and secretion of IL-6, MIP-1α and CCL2 on connective tissue and mucosal BMMCs [71]. In contrast, another study demonstrated that LPS did not affect degranulation response, however it increased FcεRI-induced cytokine production in BMMCs and MC/9 cells [50].

#### Negative Control of TLR4 Signaling System in MCs

Immune responses induced by TLR4 receptor triggering are essential for protective mechanisms against Gram-negative bacteria and other insults [17,23,56], however, constant activation of this receptor may also contribute to chronic inflammatory disorders [72]. Studies developed in MCs led to the finding that activation of some receptors can inhibit TLR4-induced cytokine production and secretion by altering its signaling pathway. In line with this, some evidences have demonstrated that opioid stimulation of MCs prevents LPS-induced TNF-α secretion in a μ- and δ-opioid receptor-dependent manner [53,73]. By using an in vivo model of endotoxemia, morphine or fentanyl pretreatments were shown to inhibit MC-dependent TNF-α production after LPS injection in murine peritoneal cavity [53,73]. Some experiments carried on BMMCs demonstrated that morphine inhibition could be explained by a decreased TRAF6 ubiquitination and a reduced phosphorylation of important proteins of the TLR4 receptor cascade such as TAK1, IKK, SNAP23 and ERK1/2. Moreover, a novel molecular complex composed by TRAF6 and β-arrestin was shown to participate in TNF-α reduced secretion in that cellular model [53]. The cholinergic anti-inflammatory pathway has also been implicated in LPS-induced MCs activation. Stimulation of the nAchRα7 cholinergic receptor was shown to prevent TLR4-induced TNF secretion in vivo [74] as well as TNF mRNA expression and protein exocytosis in BMMCs, through the inhibition of IKK, p65-NFκB, ERK1/2, and TACE phosphorylation [54]. Another physiological molecule that has been implicated in the control of TLR4 receptor-induced responses is the purine base adenine. Using BMMCs from BALB/c mice, adenine has shown to inhibit the LPS-induced production of TNF-α, IL-6, and IL-13 by a mechanism that includes the attenuation of NFκB and AKT activation [75].

Besides the control of the TLR4 response by the activation of other receptors, one of the most striking characteristics of the TLR4 signaling system is its ability to be desensitized through a process that lead to a phenomenon known as endotoxin tolerance (ET). ET is defined as the reduced capacity of a cell to respond to LPS after an initial exposure to this stimulus. It is considered a protective mechanism to prevent excessive production of inflammatory cytokines in order to avoid tissue damage and secure homeostasis [76,77]. This phenomenon has been mainly studied in macrophages and DCs, where different mechanisms, that include the expression of inhibitory proteins (such as IRAK-M, SHIP1, SOCS-1, SOCS-3, and BCL-3 among others), have been described [78,79]. Although tolerance to LPS has been less explored in MCs, there is evidence that demonstrates the development of this phenomenon in these cells. In primary BMMCs from C57BL6/J, ET has shown to be established at 2 h [55] and seems to be maintained as long as 24 h after LPS pretreatment [64]. The latter was demonstrated with the analysis of TNF-α secretion, which was prevented in response to a second endotoxin stimulation [55,64]. In other MCs models, ET was shown to inhibit not only TNF-α secretion, but also IL-6 [59,80], IL-1β and nitrites release [59]. The mechanisms that seem to be involved in this desensitization process include the inhibition on the phosphorylation of IKK, ERK1/2 [55], NFκB, p38-MAPK [55,80] and the increase in the expression of SHIP1, IRAK-M [55] and suppressors of cytokine signaling (SOCS)-1 and -3 proteins [80], all markers of ET. Another study developed in peritoneal mast cells (PMCs) that, unlike BMMCs, are defined as connective tissue phenotype, demonstrated that B-cell lymphoma 3-encoded protein (BCL-3) is required for the effective turning-off of TNF-α expression in response to LPS, therefore, affecting ET development. This was distinct to the observed in BMMCs, in which BCL-3 did not participate on this phenomenon [76].

There is evidence obtained in macrophages that ET can be induced not only by persistent TLR4 stimulation, but also from other TLRs agonists and even TLR-induced inflammatory cytokines or lipid molecules. This effect is known as cross-tolerance, heterologous tolerance or heterologous desensitization [77]. Cross-tolerance in MCs has been demonstrated by multiple studies. Stimulation of BMMCs with LPS produces autocrine acting transforming growth factor β (TGF-β) which, in turn, induces the expression of the phosphatase SHIP1. This last enzyme converts the PI3K´s second messenger PIP3, into PIP2, thereby causing the inhibition of the TLR4-PI3K- dependent pathway [59]. As the authors of this study pointed out, expression of this phosphatase is essential for the induction of ET in macrophages, so it is possible to hypothesize that the same could happen in MCs. In line with this, a more recent study demonstrated that the endocannabinoid (eCN), 2-arachidonoylglycerol (2-AG), is also participating in the induction of LPS tolerance in MCs. Utilizing BMMCs, 2h pretreatment with 2-AG, acting in the CB_2_ cannabinoid receptor, prevented TNF-α secretion induced by LPS. This 2-AG-induced tolerance shared some molecular targets with the classical ET, such as the inhibition of IKK and p65-NFĸB phosphorylation and the induction of IRAK-M (which blocks IRAK1/IRAK4 signaling) and SHIP1 expression. In contrast with canonical ET, 2-AG did not prevent LPS-induced MAPKs phosphorylation, indicating that the eCN acts only in one branch of the TLR4 signaling pathway. Moreover, the authors demonstrated that 2-AG could act as an autocrine molecule to contribute to LPS tolerance, as it is produced in response to continuous endotoxin stimulation in MCs [55]. As described in that study, 2-AG tolerance could also have implications in the physiological control of TLR4-dependent MCs activation, as LPS-induced secretion of TNF-α was prevented with 2-AG [55] however, more studies need to be done to corroborate in vivo 2-AG-induced tolerance. Although the mechanisms by which eCNs could be synthesized in these cells in response to LPS have not been elucidated, they need to be promptly approached to understand how inflammatory reactions are self-limited in innate immunity and a promising field related to the control of innate immunity-triggered responses in MCs. 

Some other works on MCs have also included the study of epigenetic modifications during ET. That is the case of the work developed by Poplutz and cols., where they described that in tolerant BMMCs, LPS stimulation prevented the demethylation lysine 9 of the core histone H3 (H3K9) which in consequence inhibited NFĸB binding at *Il6* and *Tnf* promoters [76]. The latter indicates that epigenetic modification of chromatin and DNA may be a novel mechanism for the regulation of development, maintenance and control of ET in MCs, whose long-term consequences remain to be elucidated. 

**Figure 3:**
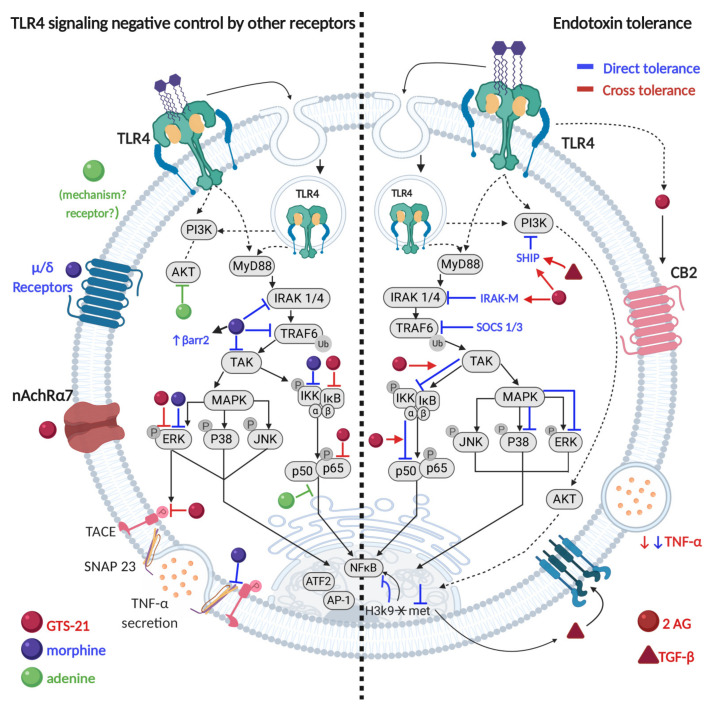
Control of TLR4-mediated signaling in MCs. Morphine, GTS-21 and adenine have been proven to control TLR4-induced cytokine production on MCs (left side). Morphine exert its effects by acting at μ/δ opioid receptors while GTS-21 (see reference [54]) inhibits LPS-dependent response through the activation of nAchRα7 receptor. Although adenine has been demonstrated to inhibit TLR4-induced responses, the receptor or mechanism by which it mediates that effect has not been described. Symbols of the distinct molecules were included in the interior of the cell to illustrate the points of TLR4 signaling pathway blockade. ET on MCs (right side) can be developed by direct (blue) or indirect (red) mechanisms. Direct tolerance depends on the increase of SHIP, IRAK-M and SOCS1/3 expression and the inhibition of IKK, p65-NFκB, p38 and ERK1/2 phosphorylation. Inhibition of the histone H3K9 demethylation also participates on this phenomenon. Cross tolerance includes the production and autocrine action of TGF-β and 2-AG. TGF-β contributes to the expression of SHIP, however, the specific activation of a receptor has not been described. By the activation of CB_2_ receptor, 2-AG induces the expression of SHIP and IRAK-M and inhibits the phosphorylation of IKK and p65-NFκB, thereby, inhibiting TNF-α production. Figure created with Biorender.com.

In conclusion, unraveling how the TLR4 receptor is activated in MCs has led to the description of novel molecules that are necessary in the TLR4 signaling pathway and that mediate its negative control during the shutting down of the receptor signaling network (Figure 3). Moreover, TLR4 triggering in MCs has shown to connect the innate immunity receptor signal transduction pathways, not only with the transcription of cytokine genes (as classically defined in other immune cells), but also with the secretory mechanisms of pre-formed mediators as well. Studies of the activation of TLR4 receptor on MCs need to continue in order to develop a better understanding of the initiation, maintenance and control of the innate immune responses against bacteria or other insults in this cell type.

### 3.2. TLR2-Dependent Activation of MCs

Similar to the TLR4 receptor described above, the TLR2 receptor is widely expressed in diverse immune cells and recognizes a wide variety of pathogenic ligands. Typically, TLR2 is considered a receptor for peptidoglycans (PGN) derived from Gram-positive bacteria, however, as it associates with other molecules such as TLR1 and TLR6 [81], it can have different specificities. Thus, depending on the heterodimer formed, TLR2 can recognize molecules with diacyl and triacyclglycerols moieties, proteins and polysaccharides. For example, the TLR2/1 complex recognizes Pam3CSK4 and triacylated lipopeptides derived from bacteria and mycobacteria, while the TLR2/6 heterodimer can detect Pam2CSK4, FSL1 and MALP2 (as synthetic ligands) and diacylated lipopeptides from mycoplasma and yeasts (zymosan) or group B Streptococcus derivatives (LTA) [66,81,82]. Those differences in ligand binding can be explained by the presence of hydrophobic pockets in the TLR1 and TLR2 receptors. The amide-bound lipid chain of the triacylated lipopeptide binds to the TLR1 while the remaining lipid chains interact with the TLR2 receptor. As this hydrophobic channel is absent in TLR6, its ligand recognition is influenced by the molecule that binds to the TLR2 receptor [81]. 

Once stimulated, TLR2 heterodimers initiate the MyD88-dependent pathway that has the same consequences as described for the TLR4 receptor. Briefly, TLR2 triggering induces the activation of the IKK- and MAPKs- dependent pathway with the subsequent production of inflammatory cytokines. Although less studied, some molecules have been confirmed to participate in TLR2 signaling in MCs. Using MC-9 cells, it has been demonstrated that participation of IRAK1 is essential for P3C [50] and PGN [51]-dependent MCs activation. Participation of IKK-dependent signaling in response to TLR2 stimulation has been demonstrated by NFκB activation. NFκB has shown to augment its nuclear expression after treatment with Pam3CSK4 on LAD2 cells [83]. On the other hand, stimulation of MC/9 cells with P3C increases this transcription factor phosphorylation and DNA-binding [50].

With respect to MAPKs signaling, both TLR2/1 and TLR2/6 heterodimers have been shown to activate these proteins in different MCs models. In BMMCs, p38 was phosphorylated in response to P3C (TLR2/1 ligand) or FSL-1 (TLR2/6 ligand) [58]. This is consistent with what has been described in MC/9 cells, where P3C induced p38 phosphorylation and JNK activation at 30 and 60 min [50]. On the other hand, participation of ERK in response to TLR2 activation has shown different results. In MC/9 cells, this MAPK did not get phosphorylated in response to P3C stimulation, however inhibition of ERK with PD98059, partially prevented TNF-α secretion in response to the same stimulus [50]. In contrast, using different human mast cell lines, LTA [35], PGN [35,84] and Pam3CSK4 [83] resulted in robust ERK phosphorylation [35], while in BMMCs, the latter also induced ERK activation in a TLR2- and MyD88-dependent manner, an effect that was shown to be essential for eicosanoid production [85]. A more recent study measuring IL-8 production in LAD2 human cells, described that JNK could be involved only in PGN- (TLR2/6) induced cytokine secretion while ERK seemed to be important in TLR2 responses regardless of the heterodimer that is activated [86]. Transcription factors activated in response of TLR2/MAPKs pathway were analyzed by Qiao and cols. in MC/9 cells, where ATF2 phosphorylation was shown to be increased in response to P3C. In contrast, c-Jun and c-FOS, were only slightly activated by the same stimulus [50].

The induction of PI3K/AKT signaling in response to TLR2 stimulation in MCs has not been completely elucidated. While in MC/9 cells AKT was not phosphorylated in response to P3C exposure (even after 60 min) [50], in LAD2 cells Pam3CSK4 increased AKT activation after short stimulation [83]. This could suggest that PI3K signaling may depend on the cell model under study, however, a more recent work developed also in LAD2 cells, demonstrated that PI3K signaling does not participate in Pam3CSK4-induced MCs responses. Moreover, the same authors described that PI3K is essential for TLR2/6 heterodimer-induced LAD2 responses, as the inhibitor wortmannin prevented PGN-induced IL-8 secretion [86]. In concordance with this, PGN was also shown to induce AKT phosphorylation in HMC-1 cells [84], demonstrating that activation of this molecule shows more consistent results when the TLR2/6 heterodimer is activated. These contrasting results have not been completely explained and need further study to characterize the existent differences.

Calcium signaling has also been observed with some TLR2 ligands and activation of the diverse TLR2 heterodimers seems to require the function of this signaling pathway in particular ways. Although incubation of LAD2 cells with PGN (TLR2/6 ligand) and Pam3CSK4 (TLR2/1 ligand) induced both a slight and transient increase of intracellular Ca^2+^, just TLR2/1 heterodimer activation has demonstrated to require this calcium rise to provoke cytokine secretion as measured with IL-8 release [86]. Nevertheless, activation of NFAT, a transcription factor that is activated in response to calcium/calcineurin signaling, has not been deeply studied. In MC/9 cells, NFAT has demonstrated to fail binding to DNA in response to P3C [50], which supports the idea that activation of Ca^2+^ signaling needs further studies in MCs.

Stimulation of MCs through TLR2 heterodimers led to the identification of non-canonical molecules in their transduction pathways. In human cord blood-derived mast cells (CBMCs), adenylyl cyclase inhibition was demonstrated to decrease IL-6 secretion in response to PGN and Pam_3_CSK_4_ [87] highlighting its importance in TLR2 receptor signaling. Moreover, Pam3CSK4 and PGN have shown to induce IL-8 secretion in LAD2 human cells in a G_o_ dependent manner, as silencing of this G protein significantly inhibited this cytokine production [83]. MCs have also shown to produce nitric oxide (NO) and nitrites in response to TLR2 stimulation. The latter was demonstrated by incubating BMMCs from C57BL6/J with zymosan or *C. albicans*. This effect was shown to be dependent on TLR2/Dectin-1 (a C-type lectin receptor) heterodimer, demonstrating an important function of this complex formation against fungi [88].

Activation of TLR2 receptor has shown to induce MCs degranulation in some studies [32,40,84,89], however, in other experiments this effect was not demonstrated [25,28,50,86]. TLR2 also induces secretion of a large variety of inflammatory mediators that present a particular profile depending of the heterodimer that is activated. These mediators include cytokines, chemokines, leukotrienes and other eicosanoids.

Tolerance of TLR2-induced responses in MCs has received less attention in comparison to endotoxin tolerance. However, there is evidence that LPS induces cross-tolerance to the TLR2 agonist P3C in these cells. Using BMMCs, LPS pre-treatment for 18 h, prevented P3C-dependent production of TNF-α and IL-6 [80]. This suggests that both receptors share molecules of their signaling pathways and, therefore, their responses may be controlled or prevented by similar mechanisms (such as the induction of SHIP, IRAK-M and molecules that act in an autocrine manner, for example, eCN).

The hypothesis of a synergistic effect of TLR2 with other innate immune receptors has been tested. Indeed, in different MC models, LTA increases the expression of the complement receptor 3 (CR3) which, in turn, enhances bacteria uptake and clearance. Moreover, MCs´ LTA pre-treatment augmented proinflammatory cytokine release after bacteria internalization [90]. In a more recent study, LTA and cathelicidin (a potential ligand of MGPRs that will be discussed later) have also shown synergistic effect by increasing the antiviral activity of BMMCs against vaccinia virus (VV) [91]. TLR2 receptor triggering has also shown to increase activation of MC-dependent adaptive immune responses. The combination of P3C, MALP-2 or PGN with antigen augments the secretion of different cytokines compared with antigen alone in MC/9 cells and BMMCs, an effect that seems to be dependent on MAPKs and the engagement of diverse transcription factors [50]. A later study also demonstrated the synergistic effect of TLR2-FcεRI receptor by the analysis of IL-6 secretion in BMMCs [92]. Interestingly, Saluja and cols. established that the time of pretreatment with TLR2/6 agonist can determine the effect on antigen-induced responses, as only long incubations with FSL-1 increased degranulation and CysLT production on mucosal and connective tissue MCs [71]. These data show that TLR2 receptor can act together with other immune receptors to enhance MCs′ responses.

It is important to understand the function of this cell type against components of different microorganisms, and to further explore the connection of TLR2 receptor activation with the secretory machinery of MCs, as has been done for the TLR4 receptor.

### 3.3. TLR5-Induced Triggering of MCs

The TLR5 receptor participates in the innate immune responses by the recognition of flagellin, the main protein component of the bacterial flagellum. Detection of flagellin is relevant because it is an important virulence factor of Gram-negative and Gram-positive bacteria [93,94]. The recognition process of the TLR5 receptor, as well as its signaling cascade has been mainly studied in epithelial cells, DCs and macrophages. Binding of flagellin to TLR5 leads to receptor dimerization [95] which, in turn, activates the cytoplasmic TIR receptor domains that lead to the triggering of the MyD88-dependent pathway. This activation induces nuclear translocation of NFκB and phosphorylation of MAPKs, therefore generating the production of inflammatory mediators [93].

Information about activation of MCs by TLR5 is scarce. Most of the existent studies have described only the production of inflammatory mediators in response to flagellin. In line with this, Kulka and cols. demonstrated that flagellin induces TNF-α and IL-1β release from human cultured mast cells (HCMCs) and LAD cells [33]; this production of cytokines could be related to the increase on ERK1/2 phosphorylation that was demonstrated in a later study carried on LAD2 cells [35].

A more recent study demonstrated that flagellin could also modulate other MCs responses. Incubation of BMMCs with flagellin induced T-cell immunoglobulin mucin domain molecule-4 (TIM4) expression, which was shown to depend on the activation of STAT6 transcription factor [96]. As STAT6 has been demonstrated to be activated by IL-4 and IL-13, it is possible to hypothesize that flagellin stimulation of MCs could induce this inflammatory mediators’ production and that these cytokines act in an autocrine way to modulate STAT6 activation. 

In conclusion, more studies about TLR5 receptor are needed as it recognizes an important virulent factor of Gram-negative and Gram-positive bacteria. Because of MCs localization in the interface between host and environment, and their ability to early release of inflammatory mediators, their response against any microorganism should be important to understand innate immune responses.

### 3.4. TLR3-Activated Signaling Pathways in MCs

Due to its participation on virus recognition, the TLR3 receptor has been widely studied on different cells, but it is less well characterized in MCs. It is an endosomal receptor that is activated in response to double-stranded RNA (dsRNA) or by the commonly used synthetic ligand polyI:C, which mimics that molecule. A specific characteristic that makes the TLR3 receptor different from the rest of the TLRs, is that on activation, it does not recruit the MyD88-dependent pathway. Instead, TLR3 signaling depends only on the TRIF adaptor protein [97]. Triggering of TRIF-dependent cascade allows signaling of MAPKs and the synthesis of different inflammatory mediators through the activation of NFκB and IRF3 [97], in a way similar to that described in the TLR4 receptor section. Activation of IRF3 transcription factor seems to be one of the most important consequences of TLR3- triggering due to the fact that it leads to the production of type I interferons, which are important for anti-viral responses.

TLR3-dependent stimulation with polyI:C induces no production of TNF-α, IL-6, or IFN-αβ in BMMCs [45]. Similar results were obtained in other study in the same cell model, where the same stimulus produced no secretion of TNF-α, IL-6 or chemokines (RANTES, MIP-1α and MIP-2) [26]. In line with the results obtained with polyI:C, the direct infection with cytomegalovirus- (CMV) has also shown that these cells may not be activated by TLR3 stimulation. By analyzing MCs from the peritoneum of CMV infected mice, it has been possible to determine that this virus induces degranulation of MCs without acting directly on this cell type but, instead, by activating the TLR3/TRIF cascade in other cells of that cavity [98,99]. In contrast, fetal skin mast cells (FSMC) which are from the connective tissue phenotype, produced TNF-α, IL-6, RANTES, MIP-1α and MIP-2 in response to TLR3 stimulation [26]. Similar results were obtained in P815, a mouse mastocytoma cell line, where influenza viruses and polyI:C treatment induced the production of different inflammatory mediators, an effect that required the participation of TLR3/TRIF activation [100]. Moreover, in rat peritoneal MCs, activation of TLR3 receptor was also detected by the production of type I interferons, cytokines, chemokines and lipid mediators [101]. These results demonstrate that either the MCs phenotype or the ligand used could influence the immune response induced by the TLR3 receptor activation.

In support to the existence of the TLR3-induced response in MCs, there is evidence of the participation of signaling molecules in response to the activation of that receptor. Specifically, the cascade formed by TLR3, IKK, and MAPKs has been studied in human MCs. PolyI:C induces the activation of IκB and NFκB. Moreover, JNK and p38 phosphorylation was also detected in response to TLR3 triggering. Both signaling cascades were demonstrated to participate, at least partially, in IFN-α production as inhibitors of NFĸB (SN50), p38 (SB202190), and JNK (SP600125) prevented its secretion [33]. A different study also showed that TLR3 stimulation inhibits the capacity of the LAD1 human mast cell line adhesion to fibronectin and vitronectin [34]. However, the signaling pathway that mediated that effect has not been explored.

Activation of TLR3 receptor on MCs has also been demonstrated by describing its synergistic effects with the FcεRI receptor. By using connective tissue like MCs (CTLMC) and mucosal like MCs (MLMC) obtained from bone marrow of C57BL/6 mice, it was determined that long incubation with polyI:C enhances antigen mediated degranulation, CysLT production, and cytokine secretion [71]. This shows that these cells can respond to TLR3 agonists and also that this receptor can interact with other immune receptors to improve MCs responses to different insults.

In conclusion, TLR3 signaling cascade constitutes an important area of study in MCs since it is the only receptor that is not coupled to the MyD88-dependent pathway. This specific feature opens an opportunity to identify the relationship between TLR3 signaling and the mechanisms that control mediator release, especially in MCs, that have the characteristic to store preformed mediators. This could help to unravel the role of MCs in the immune responses against viral infections.

### 3.5. TLR7, TLR8, TLR9 and TLR10-Dependent Stimulation of MCs

Although the study of TLR7, TLR8, TLR9 and TLR10 receptors is important to understand cell responses to different intracellular molecules, there is almost no information available of these receptors in MCs. It has been described in other cellular models that they transduce their signals through to the MyD88 cascade [66]; therefore, they should induce similar effects as the ones for the TLRs described above. 

The ligands that are recognized by TLR7, TLR8 and TLR9 have been well established. TLR7 and TLR8 are activated by single stranded RNA (ssRNA), while TLR9 detects CpG-DNA from bacteria, mycobacteria and viruses. Interestingly, TLR10 ligand has not been well described. 

Studies of TLR7 and TLR9 in MCs, are limited to the expression and induction of inflammatory mediators, while for TLR8 and TLR10, only their expression on different MCs models has been described. TLR7 and TLR9 activation on MCs has been demonstrated to induce production of Cys-leukotrienes, TNF-α, IL-6, CCL2, CCL5 and CXCL2 [26,33,34,66]. In addition, TLR7 triggering also generates IFN-β and the chemokine CCL3, while TLR9 has shown to produce IFN-α, IFN-γ, and IL-1β too [26,33,34,66].

More studies are needed to identify specific characteristics of these TLRs´ transduction pathways in MCs, since they have not been characterized in this cell model and they may constitute important targets for the treatment of different inflammatory conditions caused by intracellular microorganisms.

## 4. Nod-Like Receptors (NLR)-Dependent MCs Activation

NLRs consist in a group of cytoplasmic modular proteins characterized by a central NACHT domain composed by 300–400 aminoacids (the NOD or NBD domain) that promotes oligomerization and possesses nucleoside-triphosphatase activity. On their C-terminus, NLRs present multiple leucine-rich repeats (LRRs) that mediate ligand sensing. NLRs are divided in four subfamilies, based on the structure of their N-terminal effector domain. The NLRC subfamily is characterized by the presence of one or multiple N-terminal caspase activation and recruitment domains (CARDs) that allow direct interaction with other CARD-containing proteins. Among the NLRCs, NOD1 and NOD2 are the best characterized members and are sensors of intracellular bacterial peptidoglycan [102]. The NLRP subfamily is formed by a group of receptors that possess an N-terminal pyrin domain (PYD) and are best known for their role on the formation of inflammasomes and the induction of pyroptosis. The NLRA subfamily only includes one member, class II major histocompatibility complex transactivator (CIITA) and, finally, NLRB subfamily members contain one or several baculovirus inhibitor of apoptosis protein repeat (BIR) domains [103]. The best-described member of this family is NAIP (neuronal apoptosis inhibitor protein). 

Main signaling pathways activated by NLRs start when those molecules form large oligomers in a ligand-dependent fashion. In those aggregates (known as inflammasomes), distinct molecules are incorporated to direct signaling to NFĸB nuclear translocation, MAPKs phosphorylation and caspase 1 activation [104]. For example, the first recruited protein by NOD1 and NOD2 aggregates is a serine-threonine kinase called RICK (RIP2). Incorporation of this protein to the oligomers requires CARD-CARD homotypic interactions. RICK, in turn, is polyubiquitinated by TRAF E3 ubiquitin ligases and recruits TAK1 and NFĸB essential modulator (NEMO), that finally causes the phosphorylation of IKK and activate NFκB inducing a transcription program which is similar to that observed after TLR activation [105], promoting the expression of cytokine genes such as TNF, IL-6, IL-8 and others. RICK and TAK-1 also are involved in MAPK activation, with the participation of the adapter CARD9 [106]. Caspase 1 activation involves the participation of an adaptor protein named ASC (adaptor protein apoptosis speck protein with caspase recruitment), that is recruited mainly to oligomers composed by NLRP1, NLRP3, or NLRC4. Main function of caspase 1 is the processing of pro-IL-1β and pro-IL-18 in order to generate the mature forms of those cytokines. Incorporation of ASC to NLR complexes occurs via homotypic PYD-PYD interactions, whereas CARD-CARD interactions are needed for the caspase 1 recruitment to ASC [107]. Besides cytokine maturation, other important action of caspase1 is the induction of pyroptosis, a programmed cell death pathway that requires the formation of pores on plasma membrane formed by gasdermin oligomers [108]. Activation of NLRs triggers the initiation of a generalized inflammatory response [107].

In MCs, the expression of transcripts of several NLRs has been described. Utilizing qPCR, it was found that the human mast cell line HMC-1 express NOD2, and activation of this receptor seemed to occur in response to bacterial peptidoglycans (PGN), leading to histamine release [109]. In the mast cell line P815, NOD2 mRNA was also detected and an important induction of this messenger, together with the activation of NFκB was observed after infection with *Staphylococcus aureus* [110]. On the other hand, when human CBMCs were activated by NLR agonists alone, they did not produce detectable levels of cytokines, but NLR activation significantly increased the production of IL-6 triggered by the TLR2/1 activator Pam_3_CSK_4_ [87]. In the same cell preparation, the NOD1 ligand M-TriDAP triggered the release of IL-8, macrophage inflammatory protein (MIP)-1α, MIP-1β and TNF-α in the absence of degranulation. When cells were treated with M-TriDAP and LPS, cytokine secretion was augmented [111]. 

In BMMCs differentiated to the connective tissue phenotype (CTMCs) by culturing them with fibroblasts and in the presence of SCF, an important induction of NLRP3 transcript was observed [112]. Interestingly, NLRP3 mRNA was also found augmented after FcεRI receptor crosslinking and acted as a negative regulator of the delayed production of prostaglandin (PG) D_2_. In experiments in which NLRP3 was expressed in BMMCs, a specific blockage on cyclooxygenase (COX)-2-dependent PGD2 generation and diminished levels of COX-2 mRNA were observed, without any observable effect on degranulation, early COX-1-dependent PGD_2_ generation or cytokine mRNA synthesis. Those data suggested that NLRP3 receptor exerts regulatory functions on the FcεRI signal transduction system, specifically blocking the production of PGD2 and, potentially, affecting the onset of allergic reactions [112]. Finally, although no specific signal transduction pathways activated by NLRs in MCs have been reported, the in vivo relevance of those receptors on MCs function has started to be characterized, for example, an important increase on NOD2^+^ positive MCs was observed in colonic mucosal biopsies from Crohn´s disease (CD) patients compared to those obtained from ulcerative colitis patients or control biopsies [113], and IFNγ-treated human MCs showed an increase in NOD2 expression. Expression of NOD2 in MCs correlated with augmented TNF-α secretion after the addition of the NOD2-specific agonist muramyl dipeptide (MDP) [113].

## 5. RIG-I-Like-Dependent MCs Activation

The retinoic acid-inducible gene I (RIG-I) protein, together with the melanoma differentiation-associated gene 5 (MDA5) and the laboratory of physiology and genetics 2 (LGP2) proteins are intracellular sensors of viral RNA and constitute the RIG-I-like family of receptors (RLRs) [114]. They possess a domain called DExD/H RNA helicase domain with ATPase activity. RIG-I and MDA5 (but not LGP2) have also two N-terminal CARD domains that allow their oligomerization. RLRs recognize foreigner double-stranded RNA localized in cell cytoplasm and form large oligomers that use ATP hydrolysis to sustain their helicase activity and promote the synthesis of pro-inflammatory cytokines via NFκB nuclear translocation. BMMCs express transcripts for MDA5 and RIG-I and levels of those mRNAs were found increased after infection with the vesicular stomatitis virus (VSV) [115], and activation of those receptors leads to the synthesis of IFN-α, IFN-β and IL-6. In human CBMCs, RIG-I and MDA5 transcripts also increased after dengue virus infection [116].

Interestingly, in freshly isolated rat peritoneal MCs (PMCs) the constitutive expression of NOD1, NOD2 and RIG-1 was detected and the host defense peptides (HDPs) LL37 and hBD-2 enhanced the presence of those receptors, potentiating the pro-inflammatory and migratory responses of PMCs [117], suggesting a close interaction between innate immune environment and the capacity of MCs to express intracellular innate immunity receptors. 

## 6. C-Type Lectin Receptors in MCs

C-Type Lectin receptors (CLRs) are transmembrane proteins that contain in its structure at least one C-type lectin-like domain (CTLD) and are able to bind lipid, carbohydrate or several proteins. This family of receptors is formed by more than 1000 members, divided in 17 groups with distinct functions, like complement activation, phagocytosis, cell adhesion and innate immunity [118]. CLRs bind PAMPs expressed by bacteria, viruses, parasites, and fungi. Also, they recognize house dust mite allergens [118], tumor antigens, tumor associated molecular patterns and DAMPs. Some CLRs possess an intracellular immunoreceptor tyrosine-based activation motif (ITAMs) and after activation, they induce Syk phosphorylation, and trigger a signaling cascade that includes PKCδand the complex CARD9-Malt1-Bcl10, leading to the activation of the NFĸB transcription factor. CLRs are classified in those belonging to cluster 1 (those able to activate Syk) and those belonging to the cluster 2, which require the gamma chain of Fc receptors as adapters. Cluster 1 consists of dectin-1-like CLRs (such as DNGR1 and Clec2) and cluster 2 consists in dectin-2-like CLRs, such as MCL and Mincle [119]. 

MCs express Dectin-1 and its activation by zymosan leads to the generation of Reactive Oxygen Species (ROS) and leukotrienes [120,121]. Dectin-1 presents a structure characterized by a carbohydrate-recognition domain, a transmembrane domain and an intracellular hemi-immunoreceptor tyrosine-based activation motif (hemITAM). Tyrosine phosphorylation of hemITAM is required for the downstream activation of the tyrosine kinase Syk, which starts a cascade that culminates with the transcription of a number of pro-inflammatory and regulatory cytokines. Mincle is a CLR that interacts with glycolipids from distinct origin. Unlike Dectin-1, it lacks any recognizable domains in the cytoplasmic region able to transduce intracellular signals [122].

MCs are the predominant cell type in the skin expressing Dectin-1, which is involved in fungi recognition associated with atopic eczema [123]. MCs react against the fungi *Malassezia sympodialis* by producing cytokines and upregulating the expression of the CLRs Dectin-1 and Mincle [124,125]. Expression of Dectin-1 on BMMCs is quite low [121]. Signaling systems coupled to CLR receptors in MCs seems to be very specific and able to induce particular responses, for example, the Dectin-1 selective agonist (curdlan) leads to MC histamine release and degranulation, but, remarkably, not to the release of leukotriene C4, CCL2 or IL-6 [126]. Interestingly, it has been shown that Mincle interacts with the γ and β-subunits of FcεRI receptor and activates the tyrosine protein kinase Syk in transfected RBL-2H3 cells. Stimulation of Mincle with its specific ligand (trehalose-6,6´-dimycolate from *Mycobacterium tuberculosis*), lead to MC degranulation and PLCγ2 activation, causing ERK1/2 phosphorylation and NFAT nuclear translocation [127]. Of notice, a recent study found that Mincle expression is missing in human mast cells [29], supporting the notion that the repertoire of innate immunity receptors expressed in MC populations differ between anatomical locations and species under study. 

## 7. MGPR-Induced Activation of MCs

The MAS-related G protein-coupled receptors (MRGPRs) is a family composed of at least 38 proteins with a typical structure of seven transmembrane helixes, an amino terminal extracellular segment and the carboxy-terminal extreme located to the interior of cell cytoplasm. MRGPRs are organized in nine subfamilies designed by capital letters (A, B, C, D, E, F, G, H, and X), and individual subtypes in each family are indicated by numbers [128]. Although the search for specific ligands of MRGPRs is an active area or investigation, none of those receptors has been declared deorphanized. Ligands able to bind MRGPRs comprise many polycationic compounds, the mammalian neuropeptide FF (NFF), the neuropeptide AF (NPAF), the anti-malaria drug chloroquine, the gamma2-melanocyte stimulating hormone (g2-MSH), dynorphin 14, gamma aminobutyric acid (GABA), bovine adrenal medulla (BAM) peptides, Angiotensin 1–7, and other peptides and compounds [129]. Activation of MRGPRs has been involved in pain transmission, neurotransmission, itch, and other important physiological responses [130].

In the last years, the MRGPRX family has been found expressed and able to activate MCs in response to innate immunity stimuli. For example, the study of anaphylatoxin (C3a or C5a)-dependent activation of MCs showed that the synthetic C5a receptor antagonist (PMX-53) or the super agonist for C3a receptor (E7) required MRGPRX2 expression [131,132]. Also, cathelicidins or beta-defensins induce MCs degranulation through MRGPRX2 activation [133,134]. MRGPRX2 was proposed as the receptor involved in the effects of distinct secretagogues in the human MC line LAD2 [131,132,133,135] and, in distinct MC cellular models, MRGPRX2-induced degranulation required PTX-sensitive G proteins [131,133,134,135]. Since it has been shown that basic secretagogues are released by distinct neurons, and it has been found that the peptide hormone BAM8-22 stimulates the secretion of CCL2 chemokine in LAD2 MCs [136], it has been proposed that MRGPRX2 can modulate neuro-immune communication. On the other hand, MRGPRX2 activation by human beta defensin or LL-37 triggers a Gi/o-induced signaling pathway leading to PKC activation and a pertussis toxin (PTX) insensitive pathway involved in intracellular calcium rise [131,134].

Recently, a mouse MC-specific MRGPRB2 and its human homolog MRGPRX2 were identified as the receptors for quorum sensing molecules (QSMs), which are produced by distinct bacteria in a density-dependent fashion and contribute to virulence factor expression and biofilm formation [137]. The QSM competence-stimulating peptide (CSP)-1 triggers mouse peritoneal MCs calcium rise and degranulation in a MRGPRX2-dependent fashion, leading to MCs-induced antibacterial functions in vivo, such as protection against bacterial growth and biofilm formation [138]. Interestingly, basic secretagogues and peptidergic drugs associated with allergic-type reactions activate murine MCs through MrgprB2 and MrgprX2 receptors [139], and those effects occurred in parallel with a robust and sustained Ca^2+^ rise mediated by transient receptor potential 1/4/6 channels [140], indicating that distinct and non-canonical molecules can mediate calcium rise through MRGPR receptors in MCs. The study of MRGPRs in MCs has emerged as an active and promising field of research, leading to the development of a humanized mouse able to generate MRGPRX2-expressing human MCs for in vivo and in vitro studies [141]. 

## 8. MCs Responses Elicited by Tissue Stress 

Given the tissue distribution of MCs, the presence of pre-stored mediators in their secretory granules, and their remarkable capacity for the de novo synthesis of lipid mediators and cytokines, this cell type has been proposed as one of the first responders to tissue damage. In those circumstances, microenvironment conditions where MCs are located can dramatically change because of the generation of a hypoxic and highly oxidant niche, with the presence of damaged (necrotic) cells that release cytoplasmic components recognized as alarmins or DAMPs. Those molecules not only start a response to danger, but also are central players in tissue repair. Examples of alarmins are heat shock proteins (HSPs), IL-1α, high mobility group box 1 (HMGB1) protein, S100A/B proteins, extracellular ATP, and IL-33 [142,143]. To date, scarce information exists about the effect of hostile environment and alarmin presence on MCs effector functions or the activated signaling pathways, but important findings have been recently reported. 

### 8.1. IL-33-Dependent Activation of MCs

Alarmin IL-33 is a protein secreted mainly by necrotic cells [144] and also by epithelial cells exposed to fungus [145]. It belongs to the IL-1 family of cytokines [146] and it is the ligand of the ST2 receptor complex [147], which is composed by ST2 and the IL-1R accessory protein (IL-1RAcP) [148]. ST2 complex is expressed (mRNA and protein) in a number of immune cells, including distinct preparations of MCs [149,150,151]. Actions of IL-33 on MCs are related to exacerbation of antigen-driven airway inflammation occurring in asthma [152], and the blockage of IL-33 has been proposed as a therapeutic strategy for allergic reactions [153]. Also, IL-33-mediated MCs activation leading to macrophage mobilization related to malignant tumor growth was recently probed [154]. In BMMCs, it has been demonstrated that IL-33 induces the production of a number of pro-inflammatory mediators, such as IL-1β, TNF, CCL2 and prostaglandin D2 via ST2 receptor, with no evident concomitant degranulation [155], although, as occurred with TLR4 receptor, ST2-induced MCs degranulation has been reported in vivo [156]. IL-33 also promotes the release of cytokines and leukotriene B4 (LTB4) in BMMCs, when used as a part of an in vitro model of cell injury [157]. Interestingly, not only pro-inflammatory and intense responses have been described in response to IL-33 in MCs, since an IL-3-ST2-MCs activation axis promoted a protective environment that contributed to restoration of epithelial barrier function in a model of inflammatory bowel disease [158].

The signaling cascade triggered by ST2 receptor described in distinct cells include the activation of proteins such as the adapter MyD88, kinases IRAK1, IRAK4 and the ubiquitin ligase TRAF6, leading to MAPK (p-38 and ERK 1/2) phosphorylation [147,159]. Also, the tyrosine kinase JAK2 participates on IL-33-induced NFκB activation and the synthesis of pro-inflammatory cytokines [160]. In BMMCs, IL-33-mediated production of IL-6 and IL-13 is dependent on the p38 substrates MAPK-activated protein kinases 2 and 3 (MK2 and 3), whereas production of TNF relies on ERK1/2 and PI3K phosphorylation [161]. Despite the absence of degranulation, calcium mobilization in response to IL-33 was also detected in BMMCs and it has been proposed that a MKK3-p38 module which is upstream signaling MK2/3 and is not involved in massive calcium rise (and then on FcεRI-dependent degranulation), participates on Ca^2+^ mobilization triggered by IL-33 [161]. 

### 8.2. HMGB1-Induced Activation of MCs

Other important alarmin able to activate MCs is HMGB1, a protein that was identified in the search for endogenous endotoxin-like molecules produced after septic shock [162]. Since then, this polypeptide has been characterized and proven to possess all the properties of a classical DAMP [39], because it is released after cellular damage and induce the production of distinct pro-inflammatory mediators in immune cells. Normally, it resides in the cell nucleus acting as a DNA chaperone and facilitates nucleosome formation, transcription, replication and DNA repair. Once secreted, HMGB1 binds and activate distinct receptors. The main ones are the receptor for advanced glycation end products, RAGE and TLR4, which are expressed in immune and non-immune cell lineages. RAGE was the first HMGB1 identified receptor and their interaction was associated to neurite growth in the fetal mouse brain [163]. Distinct studies have probed the induction of cell migration, cell differentiation and pyroptosis in response to the HMGB1-RAGE axis [164]. Interaction amongst HMGB1 and TLR4 has been reported to rely on MD-2 accessory protein, which specifically binds the disulfide HMGB1, and not other redox forms [165]. Once bound to TLR4 receptor by its interaction with MD-2, HMGB1 activates the MyD88-dependent signaling pathway, leading to the nuclear translocation of NFκB transcription factor in distinct immune cells [165]. 

Some studies have provided evidence on the HMGB1-dependent MCs activation. Few are those focused on the characterization of anti-inflammatory activities of natural products. For example, glycyrrhizin (an extract of licorice root) diminished atopic dermatitis symptoms, and inhibitory effects of that compound were also observable in the mast cell line P815, where glycyrrhizin caused the inhibition of HMGB1-RAGE interaction [166]. Also, the natural antioxidant quercetin inhibited atopic dermatitis by diminishing the HMGB1/RAGE/NFκB signaling pathway [167] and resveratrol treatment diminished the skin inflammation (and the number of infiltrating MCs), in a murine model of dust mite-induced atopic dermatitis [168]. 

Recently, MCs were found to be important for HMGB1-induced brain inflammation and MC migration to hippocampus in rats [169]. In the same study, using the MC line P815, that alarmin lead to the production of TNF and IL-1β with the concomitant activation of NFκB transcription factor, and si-RNA-dependent inhibition of RAGE prevented pro-inflammatory cytokine production activated by HMGB1.

### 8.3. ATP and Adenosine-Dependent Activation of MCs

ATP and its breakdown products, such as adenosine, are passively released during tissue injury and actively secreted via vesicles and connexin or pannexin hemichannels [170,171], or by a classical endoplasmic reticulum/Golgi secretory pathway [172]. Outside the cells, ATP is enzymatically converted to adenosine through CD39 (an ecto-nucleoside triphosphate diphosphohydrolase) and CD73 (an ecto-5´-nucleotidase) [173]. Extracellular ATP secreted during inflammation binds to the purinergic P2 family of receptors. Among these family of molecules are the ionotropic receptors P2X (P2X1-7) and metabotropic receptors (P2Y1,2,4,6,11–14) [174]. In BMMCs, ATP induces strong degranulation and the synthesis of distinct cytokine mRNAs with the same intensity than FcεRI receptor triggering does [175]. Recent evidence indicates that calcium entry through P2X7, P2Y13 and P2Y14 is involved in ATP-induced degranulation, while P2X4 potentiates the IgE/Ag-mediated response [176]. 

On the other side, adenosine is produced under stressful environment generated during inflammation and injury [177]. It is able to activate four GPCRs, named A1, A2a, A2b and A3, coupled to distinct G-proteins. A1 and A3 adenosine receptors couple to pertussis toxin (PTX) sensitive-Gi proteins, while the A2aR couples to Gs and the A2bR to Gq/G_11_ and Gs [178]. Although the expression of distinct ARs has been reported in a variety of MCs preparations, A3 receptor seems to mediate the most important actions of adenosine on this cell type [179]. Effects of adenosine on distinct in vitro preparations of MCs and several models of inflammation, asthma and cancer have been extensively reported and summarized in accurate recent reviews [179,180]. Of main interest has been the possible role that adenosine has on the interaction between MCs and tumor cells. For example, MCs-dependent adenosine production triggered by membranes of cancer cells generates an autocrine loop, in which the activation of A3 adenosine receptors lead to PI3K, Akt and ERK1/2 MAPK phosphorylation, promoting the synthesis of angiogenic and regulatory mediators, such as VEGF, IL-8, IL-6, and amphiregulin [181].

### 8.4. Hypoxic Environment and MCs Activation

Low oxygen concentration is a condition present in the tissue microenvironment generated by innate immune reactions. Hypoxia has been described in inflamed airways [182], wounded skin [183], inflamed colon [184], arthritic joints [185,186], and solid tumors [187,188], where intense inflammatory reactions are observed. Those findings have placed the signaling pathways activated by low oxygen concentration as central elements regulating the metabolism of myeloid cells during innate immunity responses [189]. Hypoxic response differs between cells of myeloid origin (such as macrophages, MCs, and neutrophils) and cells from lymphoid origin (lymphocytes). In general, myeloid cells can adapt to hypoxia, mainly through the hypoxia-inducible factor 1 alpha (HIF1α) transcription factor, carrying out several of its effector functions under this condition [190]. However, in T lymphocytes the expression of HIF1α operates as a negative regulator, inhibiting, for example, the signaling mechanisms that lead to the TCR receptor-dependent cytokine secretion [191]. Also, it is well known that the hypoxic tumor microenvironment can modify the phenotype of immune cells, such as macrophages [192].

The effect of hypoxia on MCs effector responses and signaling pathways activated by that state in this cell type are two aspects poorly described of MCs physiology. The evidence so far indicates that these cells remain viable for several days of exposure to hypoxia (1% O_2_), and it has been shown that secretion of IL-6 in human MCs is critical for maintaining their survival under this condition [193]. Production of ROS is an important event in the response of MCs to hypoxia [194] and evidence has shown that the activity of Src family kinases, such as Fyn and Lyn (which initiate signaling events leading to the production of inflammatory mediators after FcεRI receptor triggering), is regulated by ROS [195]. Accordingly, in the chemical model of cobalt chloride-induced hypoxia, it has been shown that BMMCs secrete pro-angiogenic mediators, such as vascular endothelial growth factor (VEGF) through a mechanism that require Fyn and ROS [196]. 

Evidence indicates that effects of hypoxia on MCs depend on the time of exposure, and it has been suggested the existence of a biphasic cellular response in that cell lineage. Specifically, in BMMCs, exposure to hypoxia for three hours negatively regulates the synthesis of proinflammatory cytokines involved in the immune response against pathogens (such as TNF-α), through a HIF1α-independent mechanism, while exposure to hypoxia for 24 h favors the formation of extracellular traps and reduces MCs phagocytic capacity [197]. Also, it was observed that incubation of human umbilical cord derived mast cells (CBMCs) for 24 h in hypoxia, decreases LPS-induced IL-8 secretion [193].

**Figure 4:**
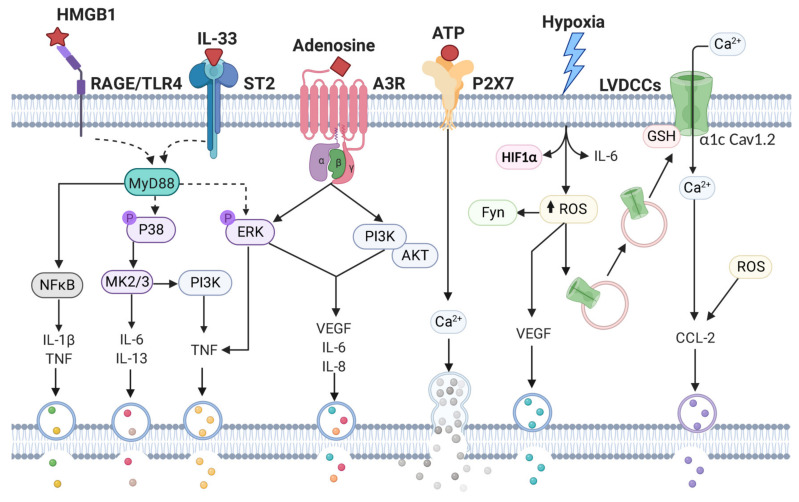
Molecular events involved in the response of MCs to mediators produced by cell damage and stress. HMGB1, IL-33, adenosine and ATP are the best-characterized alarmins able to activate MCs after tissue damage. Together with hypoxic microenvironment, those signals activate distinct signaling pathways leading to cytokine production in MCs. Binding of HMGB1 to RAGE leads to the activation of the MyD88-dependent signaling pathway leading to NFκB transcription factor and promotes the production of TNF and IL-1β. IL-33 activates ST2 receptor and also triggers the MyD88-dependent signaling pathway. Downstream effectors of MyD88 (MK2/3) are essential for the secretion of IL-6 and IL-13. In addition, IL-33 favors the production of TNF with the participation of ERK and PI3K. ATP activates P2X7 purinoreceptors and induces calcium influx with the consequent degranulation. Activation of adenosine A3 receptor by adenosine triggers ERK and PI3K and promotes the production of inflammatory and angiogenic mediators. Hypoxia leads to the stabilization of HIF1α, production of ROS and Fyn -dependent VEGF and IL-6 secretion. ROS production promotes the glutathionylation (GSH) and translocation of the α1c subunit of Cav1.2 L-type voltage-dependent Ca^2+^ channels (LVDCCs) from intracellular pools to plasma membrane. ROS and calcium entry promote the accumulation of CCL2 mRNA to, finally, induce the secretion of that chemokine. Stimulation pathways are represented with solid lines, whereas putative pathways are shown in dashed lines. Created with Biorender.com.

In a more recent study utilizing BMMCs, it was shown that hypoxia favors HIF1α stabilization and the production of the monocyte chemoattractant protein-1 (MCP-1, CCL-2). The latter occurred through a mechanism that requires the production of ROS and an increase in intracellular calcium concentrations mediated by nifedipine-sensitive, L-type voltage dependent calcium channels (LVDCCs). In that study, it was shown that hypoxic microenvironment induces the membrane translocation and glutathionylation of the α subunit of the Cav1.2 calcium channel. Those data suggest that LVDCCs channels could be considered new players in the signaling pathways activated by hypoxia in immune cells (Figure 4). In vivo, it was observed that MCs are located preferentially in the hypoxic zones of murine B16/F10 melanoma tumors and are hypoxic too, showing that these cells can detect the decrease in oxygen concentrations in that environment. The latter was corroborated by observing MCs that were positive for the hypoxia marker pimonidazole [198] and by analyzing the stabilization of HIF1α in that cell type [199]. 

Despite the existing data, additional studies are required to understand the effect of hypoxia and hypoxia-activated signaling pathways on the production of MCs-dependent inflammatory mediators in vitro and in vivo. 

## 9. Exosome Production by MCs

Novel mechanisms driving the biological actions from MCs mediators continue to be described and include the secretion of insoluble heparin and TNF-containing granules as intact particles that can be diffused away and be transported through lymphatic vessels to exert their effects on remote lymph nodes [200]. As the depth of understanding grows it becomes evident that the evaluation of the secretory activity of MCs triggered by distinct signaling cascades would be incomplete without taking into consideration the targeted extracellular vesicle-mediated secretion. The International Society for Extracellular Vesicles (ISEV) defines “extracellular vesicles” (EVs) as particles naturally released from a cell that are delimited by a lipid bilayer and cannot replicate [201]. Heterogeneous populations of EVs containing proteins, lipids and nucleic acids, are released by eukaryotic cells and may deliver their biologically active cargo into recipient cells to elicit a functional response. EVs heterogeneity is reflected in size (ranging from 100 to 200 nm), biogenesis, cell of origin, cargo, biochemical surface composition and, as a consequence, nomenclature, that includes: exosomes, ectosomes, small EVs, medium/large EVs, oncosomes, etc. Since the characterization of these different vesicle subsets is hard due to the lack of discriminatory markers, they are collectively termed EVs [202]. The EVs capacity to trigger phenotypic changes relies on a variety of up-take mechanisms displayed by acceptor cells; however, the specific or stochastic nature of these mechanisms remains controversial [203]. 

The role of exosomes in immune responses has been a focal point of attention, as it has been suggested that EVs from both immune and non-immunes cells can orchestrate the innate and adaptive immune responses [204,205,206]. However, only very recently, the vesicular components of the MCs secretome have been specifically and excellently reviewed [207]. From these earlier works, some particular features of the bi-directional vesicular-based communication between MCs and other effectors of the immune responses were highlighted. Thus, it was demonstrated that EVs derived either from BMMCs or from established MCs lines (HMC-1, MC/9, LAD2) induce the phenotypic and functional maturation of DCs, induce differentiation of naïve T-cells to a Th2 phenotype, enhance cytokine secretion from B cells and T cells in vitro and influence gene expression in other MCs and CD34^+^ progenitor cells (reviewed in [207]). In pathological conditions such as inflammation or tumor progression a number of molecules associated to MCs-derived EVs have shown to alter target cell phenotypes, enhance proliferation and migration and modulate angiogenesis, amongst other functions [207,208,209,210]. In the context of a bi-directional communication system, it has also been described that MCs degranulation and cytokine secretion can be induced by the up-take of membrane-bearing “microparticles” released from activated T cells but not from resting T cells [211,212]; while treatment with mesenchymal stem cell-derived EVs is associated with reduced infiltration and activation of MCs both in vivo and in vitro [213]. 

In recent years, new experimental approaches are leading to a more refined and precise knowledge of the molecular mechanisms underlying MCs-derived EVs functions and biological impacts. Here we compile the latest evidence in order to provide an updated grasp of these novel instruments of MCs signaling, from two outstanding perspectives: the ever-increasing role of mast cell exosomal RNAs and the mast cell EVs breakthrough in cancer research. 

### 9.1. The Ever-Increasing Role of Mast Cell Exosomal RNAs

Since the seminal demonstration that human MCs release RNA-containing vesicles that provide them with the capacity to shuttle RNA between cells and promote directed gene expression changes [214], a strong effort has been made to elucidate the characteristics and function of the different subpopulation of secreted RNAs. Functional assays linked to microarray analysis proved that exosomal RNA content differs between exosomes derived from cells exposed to different experimental conditions and that this is associated to their biological function on target cells [215]. In those early studies, the focus was pointed to the appearance of mRNA and microRNAs in the vesicular fractions [216]. 

Recent refined analyses were able to isolate two distinct vesicle-associated RNA profiles secreted from the mast cell line MHC-1 [217]. These two fractions, which are distinguishable by buoyant density, contain distinctive RNA signatures that could be derived from different biogenesis pathways. Subtypes of RNA found in MCs derived fractions include mRNA, miRNA, lincRNA, antisense RNA, vault RNA, snoRNA, snRNA, mitochondrial rRNA, mitochondrial tRNA, tRNA, piRNA and Y RNA. Interestingly, the latest report from the same group evidences the existence of EVs subpopulations that may also vary in their DNA content [218]. One step further is represented by the use of quantitative proteomics technology and RNA sequencing to characterize protein, long noncoding RNA and microRNA signatures in extracellular vesicles derived from resting and degranulated BMMCs [219]. Although the function of the majority of the catalogued molecules still has to be delineated, the authors find enrichment of molecular players associated to the regulation of chemotaxis, cytokine and receptor-mediated signaling pathways, MCs degranulation, cell migration, tumorigenesis and metastasis. On the whole, these studies could be the foundation for novel strategies to tackle the enormous task of deciphering the role of MCs in pathological processes such as failed immune response and cancer.

### 9.2. The Mast Cell EVs Breakthrough in Cancer Research

EVs play an essential role in cell-cell communication and regulate key processes for tumor development and growth [220,221]. Moreover, the knowledge that non-tumoral cells present in the tumoral microenvironment (TME), like MCs, could release EVs has provided a major breakthrough in cancer research opening up new insights regarding diagnosis and therapeutics [222,223,224].

The evidence suggests that MCs-derived EVs play an important role in tumor development by shuttling relevant information between tumor and stromal cells. These EVs are double-edged swords that can have both pro-tumoral and anti-tumoral functions [210]. Pro-tumoral actions were described for the first time by Xiao and cols. in 2014. They demonstrated that human mast cell line, HMC-1, could release KIT protein-containing exosomes, which can be transferred to the human lung carcinoma cell line A549. Moreover, these exosomes activated KIT-SCF downstream signaling in A549 cells, enhancing their proliferation and migration by PI3K/AKT and cyclin D1-dependet mechanisms [209]. More recently, Kim and cols. isolated EVs from an antigen activated-Rat mast cell line (RBL-2H3) and found that these exosomes increased the migration and invasion potentials of melanoma cells B16F1 by an autophagic mechanism in a p62-dependent manner [225]. 

Indirectly, MCs-derived EVs may have anti-tumoral functions by inducing B and T lymphocyte maturation and proliferation [226], enhancing the differentiation of naïve CD4^+^ T cells toward Th2 cells [227], and inducing phenotypic maturation of DCs which display a potent Ag-presenting capacity to T cells [228]. These findings suggest that EVs secreted by MCs could represent a key component of immunoregulatory network in the TME to activate and recruit immune cells that can fight against tumor cells during the early events of cancer.

On the other hand, the opposite communication has also been described, that is, from tumor cells-derived EVs toward MCs. Diverse studies have demonstrated that EVs release from diverse cancer cells lines can be taken up by MCs and trigger downstream signaling that lead to MCs activation [229,230,231]. One of these studies showed that EVs from A549 cell line induces MC-activation through SCF-KIT signal transduction, leading to release of tryptase, a protease stored in MCs granules. In turn, MCs-derived tryptase can enhance proliferation and migration of endothelial cells through the JAK-STAT signaling pathway, which is related to angiogenic processes and tumor metastasis [229]. In sharp contrast, a study reported that binding of MCs-derived tryptase to DNA-coated exosomes released by melanoma cells can be taken up through endocytosis into the nucleus of human melanoma cells where tryptase suppresses their proliferation inducing the degradation of structural and RNA-regulating proteins [232]. This suggests that MCs-derived tryptase could have an anti-tumoral contribution in melanoma. Furthermore, both studies highlight the dual participation that MCs play in tumor development and progression.

Additionally, other studies found that EVs derived from pancreatic and lung cancer cells induced MCs activation through the phosphorylation of extracellular signal-regulated kinase (ERK) 1/2. Activated LAD-2 human MCs enhanced their migratory ability and the release of TNF-α and MCP-1/CCL2 chemokine, which promote an inflammatory status and immune cells recruitment in the TME [230]. Moreover, Gorzalczany et al. found that MCs-activated by cancer EVs elicited the upregulation of genes involved in tissue remodeling and angiogenesis, including VEGF, IL-8, IL-6, and amphiregulin, a ligand of Epidermal Growth Factor Receptor (EGFR) related to lung cancer progression. Interestingly, they also demonstrated that ERK 1/2 phosphorylation was significantly reduced by an adenosine A3 receptor inhibitor and the CD73 ectoenzyme, that catalyzes the production of adenosine in the presence of ATP. This suggests that adenosine, a well-known immunosuppressive and angiogenic factor, plays an important role in the MCs activation and subsequently in the upregulation of these tissue remodeling genes [231]. The fact that MCs upregulate these genes would imply that such genes should be translated and released as soluble proteins, to perform their actions in the tumor cells. However, one exciting hypothesis provided in this study is that these mRNAs could be packaged and released in MC-derived EVs, supporting the idea that there is bidirectional communication between MCs and EVs-mediated cancer cells within the TME and that MCs can release EVs-containing functional RNAs. 

Despite the information provided by these exciting works, there is scarce information regarding the mechanisms that trigger the release of EVs by MCs. In some specific cases, pretreatment of MCs with IL-4 or IFN-α was required for EVs secretion [226,233]. However, in most studies, EVs were released at MCs resting state. It is worth highlighting the fact that in all studies, a complete culture medium with fetal serum and some MCs-growth factors, like SCF or IL-3, was used. Future studies are also needed to understand stimuli and mechanisms by which MCs release EVs and the mechanisms by which specific conditions such as inflammation or hypoxia induce and modify EVs secretion. However, we do not preclude the idea that MCs can release granule remnants or even their secretory granules (SGs) as intact particles upon strong activation leading to their degranulation [200]. Moreover, it has been reported that these granules can be taken up by other cells such as neurons, DCs, and macrophages, indicating another novel mechanism of communication between these cells [68,234,235].

**Figure 5:**
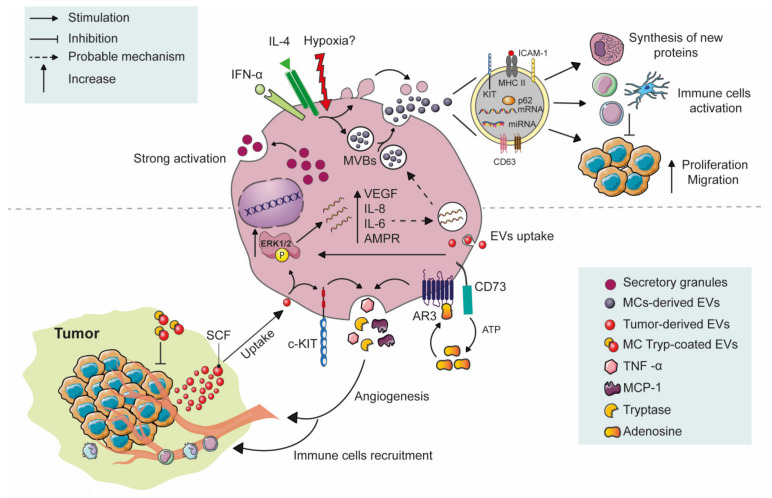
Mast cell signaling via EVs. The release of EVs by MCs could be triggered either by ligands such as IL-4 or IFN-α or tissue conditions such as hypoxia. After stimulation, EVs could be generated by budding of the plasma membrane or by the formation of multivesicular bodies (MVBs), necessary for their subsequent release. EVs carry several cargos, including functional RNAs, proteins like MHC-II, ICAM-1, KIT, p62 and, the EVs marker CD63. Transfer of functional RNAs (miRNAs and mRNAs) to other MCs can stimulate the synthesis of new proteins. Other cargos like MCH II or ICAM-1 can induce maturation and activation of immune cells, which could inhibit tumor cell proliferation. MCs-derived EVs containing KIT or p62 proteins can stimulate proliferation and migration from tumor cells (upper panel). On the other hand, tumor-derived EVs can be taken up by MCs and trigger their activation through c-KIT/SCF signaling leading to tryptase release, which can promote the angiogenic process, or it can bind to EVs released by tumor cells and inhibit their proliferation and expansion. Moreover, MCs uptake of EVs can induce an increase in ERK1/2 phosphorylation and, subsequently upregulation of genes such as VEGF, IL-8, IL-6, and amphiregulin (AMPR). It is possible that these genes can be packed into EVs by unknown mechanisms. Finally, EVs can induce TNF-α and MCP-1/CCL2 release by an adenosine and CD73 ectoenzyme dependent mechanism. TNF-α and MCP-1 mediators can induce a pro-inflammatory status and recruitment of immune cells to the tumor, respectively (lower panel).

Figure 5 summarizes the effects that MCs-derived EVs have in other cells, and the effects of cancer cells-derived EVs on in MCs. The stimuli that could lead to EVs release by MCs and signal transduction pathways triggered by EVs uptake are emphasized. Taken together, these works provide a novel way of cross talk between MCs and tumor cells via EVs and opens up the possibility of deciphering the consequences of MCs activation in the TME and their importance in the tumor biology. Very promising issues rise in the horizon. EVs may be valuable in cancer gene therapy because they could be considered safe vehicles for drug delivery due to their non-immunogenic characteristic. In that sense, understanding the anti-tumoral effects of MCs-derived EVs may hold relevant keys for therapy. But, somehow unexpectedly, other pathologies may also benefit from MCs EVs studies. It was less than 15 years ago, that Smalheiser draw attention to the “likelihood that exosomal signaling is a fundamental mode of communication within the nervous system” [233]. Since then, more than 700 articles, solidify the notion that EVs-mediated intercellular interaction is a novel form of non-synaptic communication in the central nervous system. This knowledge now supports an emerging paradigm-breaking concept: EVs could be crucial mediators of intercellular communication under pathological conditions such as neuroinflammation or neurodegenerative diseases. The EVs-mediated bidirectional interaction between astroglia, microglia, and neurons and their functional consequences in pathology have been explored and revised elsewhere [236,237]. Nascent evidence is revealing the role of MCs-derived EVs in the nervous system [238]. Our prediction, and certainly our hope, is that the near future will hold and exponential degree of knowledge in these areas of research. 

## 10. Mast Cells: Sensors of Discontinuity and Initiators of Change

Through their activation by PRRs in the initial response to infection and tissue damage, MCs importantly participate in the three levels of activation and regulation that maintains homeostasis of the organism: (1) the activation of the immune system by discontinuities induced by microbes, tumors and injuries; (2) cross-regulation of immune responses to distinct types of discontinuities; and (3) global regulation of the immune system within the organism [239]. Current hypotheses propose that this position of privilege is assured by the expression of a particular array of PRRs and signaling pathways established by the influence of locally produced mediators that model the phenotypic characteristics of bone marrow-derived progenitors recruited to distinct tissues [240,241]. However, recent evidence suggesting that definitive MCs progenitors populate skin and expand locally to form clonal colonies covering stable territories, independently of bone marrow contribution, has challenged that concept and opened the interesting possibility that the gene expression profile of PRRs in this cell type could also depend on the clonal origin of certain MCs populations, at least, in selected niches [3,4,5].

Accordingly with their important role as tissue integrity sensors, signal transduction pathways triggered by microbe products and DAMPs via multiple innate immunity receptors occurs rapidly and, in contrast with the existing model of PRR activation in macrophages, microglia and dendritic cells, signaling pathways activated by those receptors in MCs are connected not only to the transcription of cytokine genes but, importantly, to the production of pre-formed mediators, which seems to be secreted mainly by piecemeal degranulation. Activation of canonical NFĸB-regulating proteins described for TLR4 or FcεRI signaling cascades (such as IKK) leads to the phosphorylation of components of the secretory machinery (such as SNAP23), facilitating exocytosis. Also, activation of ERK1/2 MAPK controls not only the nuclear translocation of transcription factors (such as ELK-1 or c-FOS), but also the proteolytic processing of TNF-α. On the other hand, dynamin-dependent internalization of TLR4 (which is involved in IRF transcription factor activation in macrophages), is needed for ERK1/2 activation in MCs and Huntingtin, that seems to negatively regulate NFĸB signaling in monocytes, participate in TLR4-dependent synthesis of TNF-α, IL-10 and TGF-β mRNAs in this cell type. 

Remarkably, signaling pathways triggered by TLR receptors, nAchRα7 or Gi-coupled receptors (such as the opioid and cannabinoid GPCRs) promote tolerance in MCs, diminishing the production of pro-inflammatory mediators stimulated by LPS. To date, no specific element has been identified as the potential common mediator of tolerance, although some candidates have been proposed, such as the scaffolding protein β-arrestin [53] or the Janus tyrosine kinase JAK2 [54]. On the other hand, synergistic activation of MCs has been associated to the capacity of several ligands to induce the engagement of MAPK [50] or to promote calcium mobilization. Relevance of the described signaling cascades involved in innate immunity responses will be certainly found in the increasingly complex actions of MCs controlling the tolerance to skin microbiome [242] and modulating the influence of intestinal bacteria on neurological and psychiatric conditions [243,244].

Participation of MCs on the cross-regulation of immune responses among distinct cell types leading to global immune response regulation is observed in the influence of MCs-produced mediators on other cells located even far from the site of their activation. Exosomes produced by MCs control tumor angiogenesis, together with proliferation and migration of distinct immune cells. Interestingly, secretion of exosomes has been characterized mainly in resting conditions, indicating that secretion of those particles is included in the intrinsic sentinel functions of MCs, and that signaling pathways activated by maintenance factors, such as IL-3, could be involved in the basal secretory phenotype of this cell type. 

Studies on the initial innate-immunity activation and posterior tolerance on this cell type indicate that, as classic innate immune cells, it responds to change but becomes adapted when the change is long-lasting, as predicted by the discontinuity theory of immunity [245]. Remarkably, MCs emerge as a cellular model to precisely analyze the mechanisms of sensing and adaptation to microenvironmental changes associated to chronic diseases, for example, continuous stimulation of TLR4 receptor leads not only to the well-described synthesis of intracellular negative regulators of the signaling pathway leading to NFĸB activation, but also to the production of extracellular lipid regulators, such as endocannabinoids and other bioactive lipids, that generate autocrine and (potentially) paracrine loops to slow down inflammation. Also, long-lasting hypoxia induces membrane translocation and activation of LVCCs, together to their glutathionylation, demonstrating the generation of discrete molecular changes induced by the continuous presence of low oxygen concentrations whose long-term consequences on MCs function remain to be elucidated. Finally, the capacity of MCs to detect discrete changes on alarmins, bacterial products or small polycationic molecules adds to the capacity to this cell type to sense its environment and, potentially, set a threshold for activation depending on the particular conditions where they reside.

Of main interest is to study the effect of long-lasting stimulation of innate immunity receptors in MCs and the sequential activation of those receptors depending on the localization of that cell type in a given damaged tissue, in a given time. Future research will give light on the physiologic consequences of chronic stimulation of MCs on the shaping of signaling pathways responsible for systemic immune reactions.
